# Individual Differences in Cognitive Constructs: A Comparison Between American and Chinese Culture Groups

**DOI:** 10.3389/fpsyg.2021.614280

**Published:** 2021-06-17

**Authors:** Gaojie Fan, Krista D. Carlson, Robin D. Thomas

**Affiliations:** ^1^Center for Computation & Technology, Louisiana State University, Baton Rouge, LA, United States; ^2^Department of Psychology, Miami University, Oxford, OH, United States

**Keywords:** cultural difference, Chinese, American, individual difference, working memory, handedness, style of processing

## Abstract

Previous studies on human cognition show that people with different cultural backgrounds may differ in various ways. However, there are other unexplored possibilities for cultural differences including degree of handedness thought to reflect hemispheric coordination, reliance on verbal versus visual representation in problem solving, and working memory capacity both spatial and operational. We assessed each of these using the Edinburgh scale, a validated scale of style of processing, and two automatic working memory span tasks. Participants were either native Chinese students (who spoke Mandarin) or American students. Data showed that culture impacted the set of measures but gender did not and these factors did not interact. Chinese and American students showed the largest difference in their operational working memory. We also examined the pattern of correlations among the measures across the two groups and found differences due to cultural group as well.

## Introduction

Variety in cognition caused by different cultural backgrounds has emerged as an important research topic (for a recent review see Na and Chan, 2015) with the work of Nisbett and his colleagues regarding cultural differences between East Asians and Westerners being among the more influential frameworks (e.g., Nisbett and Masuda, 2003). Nisbett and colleagues suggest that East Asians think holistically, paying more attention to the environment or object background and attributing causality to it. By contrast, Westerners focus more on the object itself and follow formal logic in their understanding and categorizing, processes collectively referred to as analytic cognition (Masuda and Nisbett, 2001; Nisbett et al., 2001). Research also shows other differences between these two cultural groups, including but not limited to language representation (Schmitt et al., 1994), verbal information processing (Hu et al., 1990), unconscious perceptual learning of global and local information (Kiyokawa et al., 2012), etc.

### Hemispheric Specialization

Dichotomous descriptions of differing processing approaches appear across several categorical distinctions besides cultural groups. For example, researchers of the functional asymmetry of the human brain argue that the right hemisphere deals preferentially with global information while the left hemisphere prefers local detail (e.g., Ivry and Robertson, 1998; Yamaguchi et al., 2000). More recent suggestions are that interactions across hemispheres facilitate integrative processes (Prichard et al., 2013). Another venue that features a similar contrast is research concerning cognitive styles that distinguishes between analytic/holistic and/or visual/verbal information processing (Riding, 1997). In relevant studies, researchers tend to think of analytic and verbal as one side of a dimension, while holistic and visual occupy the other (Kozhevnikov, 2007).

### Handedness

One proxy proposed for indexing hemispheric specialization was handedness, a measurement of which hand people prefer to use when performing some tasks. Early ideas believed that hand dominance reflected cerebral dominance and, hence, processing style (Springer and Deutsch, 1985; Hellige, 2001). More recently, however, researchers have rejected this simplistic idea in favor of hemispheric integration or coordination with special attention given to the role of the corpus callosum in this endeavor (Clarke and Zaidel, 1994; Schlaug et al., 1995; Bloom and Hynd, 2005; especially Witelson, 1985). The central tenet here is that greater degrees of inter-hemispheric coordination and cooperation facilitate integrative thought (e.g., Kitterle et al., 1995). Correspondingly, measuring the strength of handedness rather than the direction would serve as a better indicator of the cognitive difference in both animal (Collins, 1985) and human studies (Bryden, 1987; Christman and Propper, 2010, but see Hardie and Wright, 2014). In other words, the measurement of strong versus weak/mixed handedness is a crucial indicator of cognition since consistent handedness is usually associated with decreased interhemispheric interaction. Prichard et al. (2013) review a variety of studies that show mixed handers (those who do not use only one hand for manual tasks) have an advantage in tasks that require access to right hemisphere functionality such as episodic retrieval, belief updating, risk perception, and tolerance for ambiguity and conflict. Throughout this literature, mixed handers are described as having a greater ability to “concurrently maintain contradictory mental representations” (p. 1184, Christman et al., 2009).

Degree, but not direction, of handedness, has also been found to be associated with language. Paul Broca’s research on brain and language suggested an association between the dominant side and the dominant hemisphere for language, stating that the language hemisphere is always opposite of one’s dominant side (as represented by the dominant hand). However, later studies showed that Broca’s rule is not always correct. In a review by Josse and Tzourio-Mazoyer (2004), they discussed the findings that left-handers are more likely to have an atypical representation of language areas, that is, more left-handers have their language areas located in the ipsilateral side of their dominant side (the left side) compared to right-handers’ language areas in their dominant side, i.e., the right side (Pujol et al., 1999). Followed up with their suggestion, Isaacs et al. (2006) found that the incidence of atypical language dominance is not only associated with direction of handedness, but also degree of handedness, as mix-handers are also more likely to have atypical language areas (46%) compared to strong right-handers (9%). Isaacs and colleagues were not the only ones who found an association between language and degree of handedness. Newman et al. (2014) also found that degree of handedness is correlated to sentence comprehension. Combined with the obvious difference between English and Chinese language, and the cognitive process of them (e.g., Tan et al., 2000, 2001; Kochunov et al., 2003; Siok et al., 2004; Crinion et al., 2009), we found it necessary to investigate if there is a difference between degrees of handedness of people with American and Chinese cultural backgrounds.

In the current study, we used the Edinburgh Handedness Inventory (EHI; Oldfield, 1971) to measure individuals’ strengths of handedness. We chose the self-report measure rather than direct observation because we cared more about preference rather than ability. In addition, inventories usually provide a wider range of options than lab observations. For examples, the EHI include items such as “Toothbrush” and “Striking a match (match),” which can be difficult to observe in an empirical task. Despite its old age, the EHI is still extensively used in the field of psychology, especially cognitive neuroscience, as a valid measure of handedness (Voss et al., 2010; Erickson et al., 2011; Stoodley et al., 2012; Barch et al., 2013; Frolov et al., 2017; Deniz et al., 2019).

Compared to the original EHI, we used a modified scoring method that fixed a potential problem in the original scale. The original scoring method involves summing the values of the responses and dividing this sum by the total of the absolute values of right and left choices. However, we observed that sometimes participants did not respond to a specific item if they had not actually used that artifact. In this case, using the original scoring method would count a non-response as the same as a “use both hands” response. This is problematic given that the scale measure is often interpreted as strength of handedness rather than direction (Christman and Propper, 2010). Hence, we edited the scoring method to more accurately account for non-responses. In our revised method, to avoid the simultaneous influence of scoring nonresponses in both numerator and denominator as “no preference” answers in the original function, the denominator was computed as the number of responded-to items multiplied by 2. Hence, if the participant answered all 10 questions, the denominator would be 20. For both calculating methods the final score would range from −100 to 100, with negative scores considered as left-handed and positive scores as right-handed. The absolute value of Edinburg Handedness Inventory scores yields the degree or strength of handedness. In the current study we used the new method to calculate the handedness scores.

### Style of Processing

Cognitive style refers to a relatively stable disposition favoring one type of information processing mode over others (Riding and Rayner, 1998). It is intended to be distinguished from ability though this may be difficult to do empirically (Mayer and Massa, 2003). Two dimensions of difference occupy most research: preference for visual versus verbal representation, and preference for holistic versus analytic processing. The latter distinction likely began as a dichotomy between field-dependent and field-independent perceptions (Witkin, 1971; Witkin and Goodenough, 1981). Field-dependency occurs when one’s perception of a focal element is altered by a surrounding context. In contrast, one is field-independent if the perception of a focal element is unaffected by surrounding contexts. Clear cultural differences in perceptual effects of background between Easterners and Westerners have been documented by Kitayama and colleagues in vision (e.g., Kitayama and Ishii, 2002; Kitayama et al., 2003). In these studies, Eastern participants show advantages when context and focal information have to be integrated compared to Western observers, whereas Westerners outperform their Asian counterparts on absolute judgment tasks in which the surrounding frames need to be ignored.

A more recent cognitive style theory centers on the visual/imagery versus verbal processing dimension, that is, the “verbalizer-imager” dimension (Childers et al., 1985; Mayer and Massa, 2003; Kozhevnikov, 2007; Blazhenkova and Kozhevnikov, 2009). The theoretical background of verbalizer-imager measurement can be traced back to 1971, when Paivio (1971) hypothesized that there are two ways an individual could make use of learning materials, verbal associations and visual imagery. Paivio’s dual-coding theory suggested that both pathways were used to a different extent by various individuals to process information. In his book, he described the imagery system as the system we use to analyze images and generate mental pictures, while the verbal system was defined as the language-specific system.

Among all the scales and tasks that measure one’s visualizer or verbalizer degree (Richardson, 1977; Riding and Cheema, 1991) we adopt Childers et al.’s Style of Processing scale (SOP; Childers et al., 1985). One primary reason to use the SOP is that we wanted to measure one’s preference, rather than ability, in visualizing and verbalizing. Preference is better measured by self-report inventories than direct object observation of style-related task performance, and the SOP is found to be more easily administered than other inventories that differentiate among styles of spatial processing (e.g., Blajenkova et al., 2006). Also, compared to other self-report measures whose validity or reliability has been questioned (Antonietti and Giorgetti, 1998; Cook, 2008), the SOP has been shown to be both valid and reliable (Heckler et al., 1993; Ong and Milech, 2001), even not perfectly.

### Working Memory

Both of the aforementioned dimensions, handedness and cognitive style, have at their core the notion of selectivity. Meanwhile, the ability to selectively attend to relevant features in the face of competing demands is the hallmark of effective working memory and has been found to differ considerably across individuals (Downing, 2000; de Fockert et al., 2001; Conway et al., 2005). Working memory refers to one’s ability to focus on goal-directed activity in the face of potentially distracting demands for attention (Baddeley and Hitch, 1974). The most well-known model for working memory was proposed by Baddeley and Hitch (1974). They suggested that working memory is a combination of three items: central executive, phonological loop, and visual-spatial sketchpad. Among the three of them, central executive controls attention and information processing, and phonological loop and visual-spatial sketchpad are responsible for temporarily holding verbal and visual information, respectively. While working memory enables the maintenance and manipulation of information, it is limited in capacity (e.g., Cowan, 2010). Daneman and Carpenter (1980) developed the first complex span task to measure people’s working memory capacity, specifically the component of control in which interfering information needs to be filtered while goal-relevant data maintained. That is, unlike traditional simple span tasks, complex span tasks (also called “dual tasks”) involve a secondary processing task in addition to the simple storage of an ordered sequence. In this way, participants are distracted from the information they are asked to memorize and are prevented from using their short-term storage to finish the task. Since then, researchers have created a variety of span tasks (Turner and Engle, 1989; Engle et al., 1999; Kane et al., 2004; Conway et al., 2005; Unsworth and Engle, 2007; Barrouillet et al., 2011; Redick et al., 2012), which share the same basic idea of a dual task, but use various types of to-be-remembered stimuli and concurrent distractor stimuli (Alloway and Alloway, 2013).

However, the problem of whether the working memory capacity measured by all of these span tasks is task-specific has never really been answered completely (Chow and Conway, 2015). For example, Daneman and Carpenter (1980) suggested that in order to use the measured working memory capacity to predict reading ability, the secondary task used in the protocol must be a reading task. But this is not what Turner and Engle (1989) found, as they concluded from their study that the correlation between working memory capacity measured by a complex span task and reading comprehension does not depend on the nature of the secondary task used in the complex span task. On the other hand, based on our previous discussion about verbal/analytic and visual/holistic thinking systems, complex span tasks that differ in the qualitative type of material (verbal versus visual) may produce different results from an individual differences perspective. In case of any influence from the secondary task on the working memory capacity measured, we used two complex span tasks with entirely distinguishable secondary tasks in the current study. One is operation span task (Unsworth et al., 2005) and the other is symmetry span task (Redick et al., 2012).

Even though the review of Na and Chan (2015) couches many of the cultural differences observed as one of focal versus diffuse attention, only a limited number of attempts have been made to examine the cultural difference in working memory. Ismatullina et al. (2014) used a spatial working memory task to study the difference in working memory in adolescents from Russia and Kyrgyzstan (Ismatullina et al., 2014) and found no significant difference correlated with cultural background. Imbo and LeFevre (2010) examined the argument that phonological and visual working memory are selectively involved in arithmetic problem solving as a function of operation (Lee and Kang, 2002) with Chinese- and Canadian-educated individuals. They only found this selective involvement for Chinese-educated participants but not for Canadian-educated participants. These findings suggest that phonological and visual working memory might be utilized differently by people with various cultural backgrounds, but more research on this topic is desired to determine how this difference exists. In the current study, we tried to contribute to this question by comparing people with East Asian and American cultural backgrounds on their working memory capacities.

### The Present Study

Handedness, cognitive style, and working memory have been extensively studied in Western culture in the past decades. However, whether they are consistent across cultures remains unknown. The present study intends to answer this question. In the current study, we measured handedness, cognitive style, and working memory in a sample of Chinese students and American students. Our purpose was twofold: we examine the distributions of these measures across the two cultural groups and explore the correlational structure among them overall and conditioned on cultural group. From these analyses, we hope to gain insight on how these dimensions of difference distribute across groups, how they relate to one another, and how culture may moderate that relationship. The objectives of our study are:

1. We are going to explore the cultural difference in degree of handedness. Previous research only showed that culture could play a role in the individual difference in handedness, but didn’t predict how. Whether one of Chinese and American cultures would be related to more mixed-handers or extreme-handers is to be discovered.
2. We predict that Chinese people should be more visual whereas American people should be more verbal when measured by the SOP. This prediction is supported by the holistic East Asian culture and analytic Western culture.
3. We would like to explore the group difference in working memory tasks with no predicted results. As we discussed before, there is no congruent agreement on whether the secondary task in the dual task would affect one’s performance. If complex span tasks are not task-specific, without any evidence showing that culture might cause any difference in working memory, we should see no difference in Chinese and American students in their operation span and symmetry span task performance. On the other hand, if complex span tasks are task-specific, we might see better performance from Chinese students as it could be affected by their holistic visual processing. Operation span task could be more complicated in that American students might perform better benefitting from their analytic processing style, while Chinese students’ performance might also be affected by their superior math ability (as supported by Campbell and Mandel, 1990; Stevenson et al., 1990; Whang and Hancock, 1994; Chen and Stevenson, 1995; Siegler and Mu, 2008; Byun and Park, 2012).
4. We are also going to conduct a correlation analysis. Based on the reason we discussed in 3, a correlation analysis would help us to understand better the nature of complex working memory span tasks.

## Materials and Methods

### Participants

One hundred and eighty-seven participants from Miami University participated in the study, of which 97 were born in the United States (hereafter referred to as the American sample) and 90 were born in mainland China (hereafter referred to as the Chinese sample). Data were kept for analysis only if the participants answered all questions in all inventories and completed the working memory span tasks. With this exclusion criterion, we obtained complete data from 146 participants, 79 American (22 male and 59 female, 19.08 ± 1.20 years, Mean ± SD) and 67 Chinese (22 male, 45 female, 22.99 ± 4.56 years, Mean ± SD). Among all the American students, four were African-Americans and 75 were non-Hispanic White or European American. None of the American students identified themselves as international students, and all of the Chinese students identified themselves as international students. All of the Chinese students identified themselves as belonging to one of the Chinese ethnic groups (e.g., Han, Zhuang, or Man). All American participants were native English speakers and all Chinese participants were native Mandarin speakers.

### Materials

Instruction and the images for the verb-generating task were presented to the participants on a computer screen, as were the instructions and stimuli for the working memory capacity task. The questionnaires were paper-based. Upon their arrival, the participants were asked about the language they are most comfortable with. These instruments as well as the instructions were presented in English to the participants whose native language was English and in (Mandarin) Chinese to those whose native language was Chinese. All 97 United States-born participants completed the measures in English and all 90 Chinese participants completed the measures in Chinese. The translation procedure used to create the Chinese materials followed the backtranslation method introduced by Brislin (1970). First a native Chinese speaker translated the original English versions into Mandarin, and then a second Chinese speaker translated this version of questionnaires back into English. In order to avoid contamination, the second translator was not exposed to the original version before translating. The final step required a new rater to compare the backtranslated version to the original to evaluate for possible inconsistency.

We used a modified version of the original Style of Processing scale (SOP) where validity and reliability of the scale were further affirmed (Childers et al., 1985; Heckler et al., 1993; Ong and Milech, 2001). In this 20-question scale, 10 questions were verbal-related and the other 10 were visual-related. Some sample verbal-related questions were, “I enjoy work that requires the use of words,” and “I can never seem to find the right word when I need it” (the latter question was reverse-scored). Some sample visual-related questions were, “There are some special times in my life that I like to relive by mentally picturing just how everything looked,” and “My thinking often consists of mental “pictures” or images.” The participants were asked to rate each statement from 1 (“strongly disagree”) to 5 (“strongly agree”). The final score ranged from 20 to 100, with higher score indicating more visual processing style and lower more verbal.

The Edinburgh Handedness Inventory (Oldfield, 1971) measures whether people prefer to use their right or left hand, and how strong that preference is on a variety of manual tasks. We dropped items from the original version that are no longer commonly used, such as Rake or Broom, and items that are culture-specific, such as Golf Club or Cricket bat. In our revised inventory, we provided 10 common actions and asked participants to indicate which hand they prefer to use in order to perform those actions, sample items include “Writing,” “Throwing,” and “Toothbrush.” The answer to each action ranges from −2 to 2, with 2 indicating always use the right hand and −2 indicating always use the left hand. A score of 0 indicates the use of both hands equally to perform the specific action.

**FIGURE 1:**
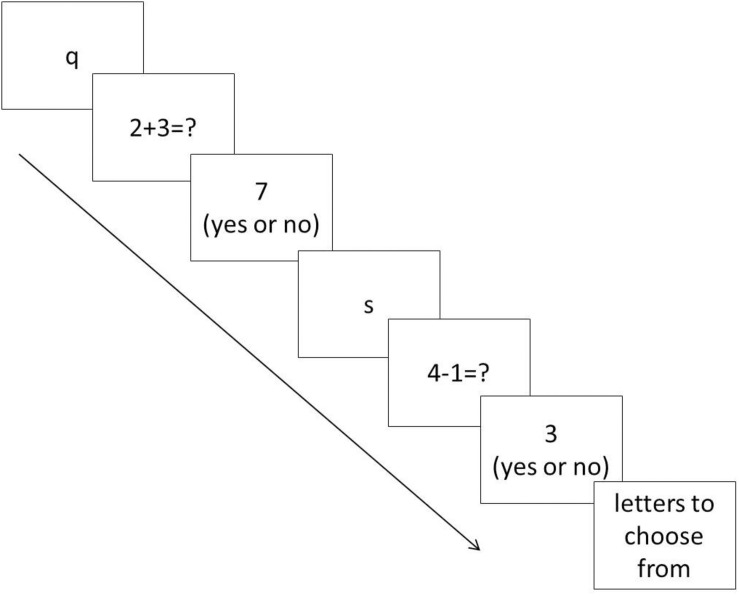
Sample process of operation span task (OSpan).

**FIGURE 2:**
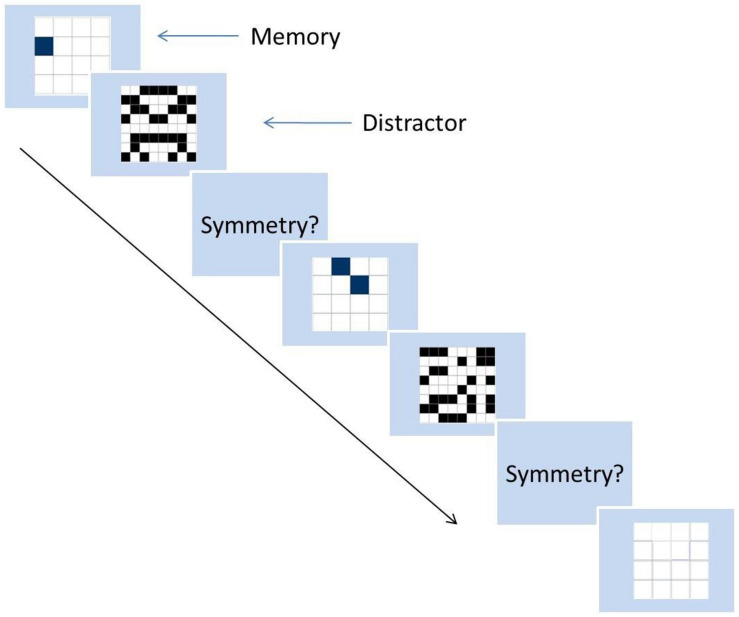
Sample process of symmetry span task (SSpan).

In the second session of this experiment, participants were asked to complete two working memory span tasks: operation span (OSpan) task and symmetry span (SSpan) task produced by the Engel lab (Unsworth et al., 2005). During the OSpan task, a series of letters each following a mathematic operation was presented to participants. Participants were required to remember the order of letters and at the same time, calculate the answers of the operations and compare their answers to a given answer then decide if the given answer is correct or incorrect. After a certain number of these trials, a group of letters was presented on the screen for the participants to choose and label the order of what they had seen (Figure 1). In the SSpan task, participants needed to remember location of squares and make decisions about symmetry. They were first asked to remember the locations of specified squares in grids and later asked indicate where these locations were, in the correct order, on a blank grid using the mouse. Following that participants needed to judge if some patterns on grids were symmetric (Figure 2).

### Procedure

After providing informed consent, all of the participants completed a verb-generation task for a different study whose data will not be analyzed here and a set of questionnaires: a demographic survey, the Edinburgh Handedness inventory, and the Childer et al.’s Style of Processing inventory in the first session of the experiment. In the verb-generation task, images of sixty-three different objects were used. Each participant was presented with twenty-one of the images and was asked to list the uses of the objects. The twenty-one objects were shown again for the participants to provide their names. These two tasks were followed by the Style of Processing inventory, the Edinburgh handedness inventory and a demographic questionnaire.

Working memory capacity was tested in the second session using Operation Span (Unsworth et al., 2005) and Symmetry Span tasks (Kane et al., 2004). This session was designed to be completed a couple of days after the first session in order to avoid the influence of fatigue. Each participant went through both OSpan and SSpan tasks, but the order of the tasks was counterbalanced so that half of the participants finished OSpan task before SSpan while the other half did the other way.

## Results

Scores for the handedness were calculated using the following formula:

(1) $$N\,e\,w\,H\,I=\frac{\sum_{i=1}^{20}X_{i}}{y}*20$$

*y* ∈ *Z*^+^, X_*i*_ ∈ *Z*, *NewHI* ∈ *Q*,

0 < *y* ≤ 20, −2 ≤ X_*i*_ ≤ 2, −100 ≤ *NewHI* ≤ 100

where *X_i_* is the answer participants gave to item *i*, and y is the total number of items answered. This score is named as NewHI because we used a different equation than the one used in Oldfield (1971), where the strength of preference was not accurately reflected, and missing data due to nonresponses were not handled ideally. In order to both reduce the negative skew common with the metric and focus on the strength of handedness, we took a natural logarithm of the absolute value of NewHI and named it LogAbsNewHI.

### The Style of Processing as Two Dimensions

Recall that the Style of Processing inventory contains two types of questions: those that address verbal materials and those that concern visual information. While scoring the Style of Processing in the manner as directed by its developers, we observed that subtotals on the two types of items were unrelated, [*r*(144) = 0.11, *p* = 0.19]. We found an acceptable internal consistency among all the items in the original scale (Cronbach’s α = 0.644), but the consistency increases in both subscales after splitting the items by question types (Cronbach’s α = 0.681 for verbal and.771 for visual).

In their 1985 article, Childers et al. suggested to treat the preference for a style of processing as a single variable, with visual and verbal processing as two directions on the same scale. As an example they proposed in their paper, people with high scores for both verbal and visual questions will be placed in the middle of the preference scale, as being fond of both verbal and visual processing indicates not having preference for either of the two directions. This interpretation made sense given the purpose of their research. For Childers and colleagues, the main goal of the Style of Processing scale was to use it in marketing applications to predict consumers’ behavior, thus it is reasonable to assume that people in the middle of the continuum, no matter whether they are high in both verbal and visual preferences or low in both, would make similar choice decisions concerning the format of materials. In contrast, our purpose is to relate cognitive style constructs to other individual difference measures, so there is no reason to assume *a priori* that people who like both visual and verbal information processing are exactly the same as people who do not like either.

Childers et al. (1985) did not assess if these two subscales were correlated or not in their sample, and they also admitted that “the empirical value of computing separate component scores is… an issue worthy of further research.” For the reasons mentioned above, we separate the overall SOPTotal into two scales: SOPVerbal and SOPVisual.

**FIGURE 3:**
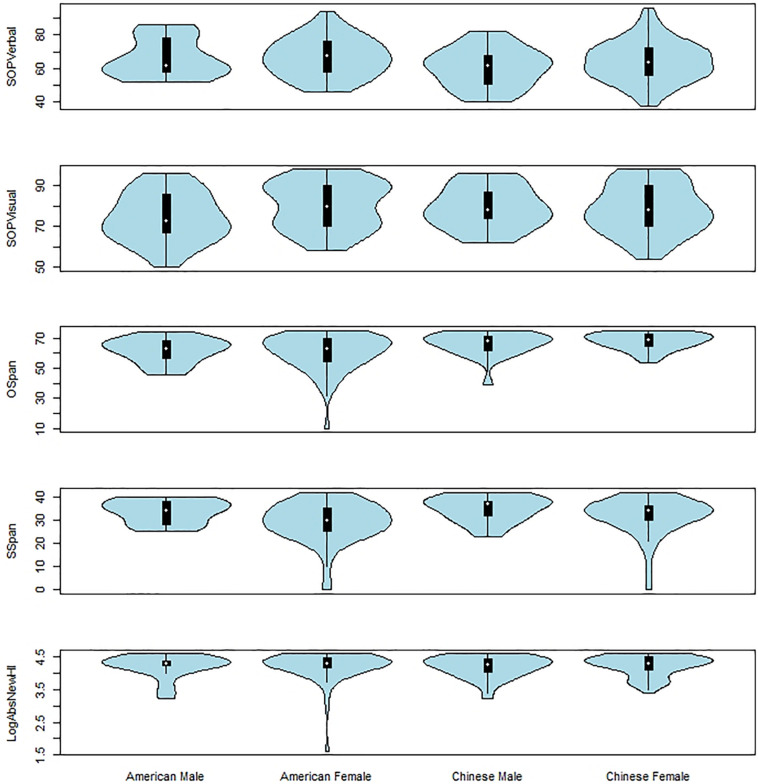
Violin Plots of SOPVerbal, SOPVisual, OSpan, SSpan, and LogAbsNewHI as a Function of Group and Gender.

With this modification for the SOP, we present summary statistics for all of the measures across each of the two cultural groups in Tables 1, 2. We also present the distributions of data, the interquartile range, as well as the medians in each culture ^∗^ gender group using Violin plots in Figure 3.

**TABLE 1:** Mean, Minimum, Maximum, and Standard Deviation Values for Each of the Five (SOPVerbal and SOPVisual for SOP) Individual Difference Measures of American Participants.

Measure	Mean	Minimum	Maximum	Standard Deviation
SOPVerbal	66.58	46	94	11.28
SOPVisual	78.66	50	98	11.73
OSpan	61.09	10	75	11.15
SSpan	30.33	0	42	7.91
LogAbsNewHI	4.22	1.61	4.61	0.43

**N* = 79*

**TABLE 2:** Mean, Minimum, Maximum, and Standard Deviation Values for Each of the Five (SOPVerbal and SOPVisual for SOP) Individual Difference Measures of Chinese Participants.

Measure	Mean	Minimum	Maximum	Standard Deviation
SOPVerbal	63.01	38	96	11.73
SOPVisual	78.57	54	98	11.29
OSpan	67.43	39	75	6.47
SSpan	32.70	0	42	7.45
LogAbsNewHI	4.22	3.22	4.61	0.32

**N* = 67*

### Comparison of Cultural Groups on Measures

The first statistical question we ask is: do the measures, as a vector, differ as function of culture and gender. To answer this question, we use multivariate analysis of variance (MANOVA) with gender and cultural group as factors, and the vector of measures as the dependent variable. We include gender as a potential predictor as there is suggestion in the literature of its influence on these constructs (Caplan et al., 1997). We found no significant interaction effect between gender and cultural group on the combined dependent variables, *F*(5, 138) = 1.07, *p* = 0.38; Wilk’s Λ = 0.963. However, we found a significant multivariate main effect from cultural group, *F*(5, 138) = 3.38, *p* < 0.01; Wilks’ Λ = 0.891. Besides that, we found a marginal significant main effect from gender, *F*(5, 138) = 2.13, *p* = 0.07; Wilk’s Λ = 0.928. The MANOVA was followed up with discriminant function analysis to see which measures contribute more to differences as a function of cultural group. Discriminant function analysis is a counterpart to MANOVA where continuous variables are used to predict a categorical variable. In our study, we used discriminant function analysis to describe how performance in the five measures could be used to predict an individual’s cultural group. Compared to *t*-test that investigates group difference for each dependent variable independently, discriminant function analysis adopts the potential relationships between dependent variables.

Since there are only two cultural groups, we obtain one discriminant function from the analysis, where canonical *R* = 0.14. This discriminant function significantly differentiated the two groups, Wilks’ Λ = 0.86, χ^2^(5) = 21.37, *p* = 0.001. The correlations between outcomes and the discriminant function revealed that OSpan loaded most highly onto the function (*structure coefficient* = 0.85), followed by SOPVerbal (*structure coefficient* = −0.39) and SSpan (*structure coefficient* = 0.38). SOPVisual and LogAbsNewHI contribute little to the function, with *structure coefficient* = −0.01 and s*tructure coefficient* = 0.01, respectively. That means OSpan working memory score will have the greatest impact of the five on the discriminant score. In other words, OSpan performance is where the two cultural groups showed the most difference out of the five measures; following OSpan, verbal processing and SSpan task performance can also explain a significant part of the group difference. The two cultural groups do not differ in their visual processing or degree of handedness.

### Examination of Correlational Structure of Measures

In this section we examine the correlational structure of the measures both overall and as a function of cultural group. We present the correlation matrix over all participants in Table 3. Besides the significant correlation between OSpan and SSpan [*r*(144) = 0.256, *p* < 0.05], we see a significant positive correlation between SOPVisual and OSpan [*r*(144) = 0.198, *p* < 0.05], and a significant negative correlation between SOPVerbal and SSpan [*r*(144) = −0.163, *p* = 0.05], which suggests that it was wise to separate SOPVerbal and SOPVisual to explore their relationships with other individual difference measurements.

**TABLE 3:** Correlation Matrix of SOPVerbal, SOPVisual, OSpan, SSpan, and LogAbsNewHI for All Participants.

	SOPVerbal	SOPVisual	OSpan	SSpan	LogAbsNewHI
SOPVerbal	1				
SOPVisual	0.11	1			
OSpan	0.06	0.20*	1		
SSpan	−0.16*	0.13	0.26**	1	
LogAbsNewHI	–0.10	0.06	0.07	−0.10	1

*p < 0.05, **p < 0.01.

The correlation matrices for each of the two cultural groups are presented in Tables 4, 5. Only in the American group did we find significant correlations: SOPVisual is highly correlated with OSpan [*r*(144) = 0.31, *p* < 0.01] and with SSpan [*r*(144) = 0.30, *p* < 0.01], respectively. SSpan and OSpan were correlated, *r*(144) = 0.27, *p* < 0.05. There were no significant correlations found in the American group.

**TABLE 4:** Correlation Matrix of SOPVerbal, SOPVisual, OSpan, SSpan, and LogAbsNewHI for American Group.

	SOPVerbal	SOPVisual	OSpan	SSpan	LogAbsNewHI
SOPVerbal	1				
SOPVisual	0.10	1			
OSpan	0.20	0.31**	1		
SSpan	–0.08	0.30**	0.27*	1	
LogAbsNewHI	–0.10	0.12	0.18	−0.10	1

*p < 0.05, **p < 0.01.

**TABLE 5:** Correlation Matrix of SOPVerbal, SOPVisual, OSpan, SSpan, and LogAbsNewHI for Chinese Group.

	SOPVerbal	SOPVisual	OSpan	SSpan	LogAbsNewHI
SOPVerbal	1				
SOPVisual	0.13	1			
OSpan	–0.05	0.01	1		
SSpan	–0.22	–0.09	0.13	1	
LogAbsNewHI	–0.10	–0.05	–0.23	−0.10	1

*p < 0.05, **p < 0.01.

## Discussion

MANOVA results showed a main effect from cultural groups, suggested that people from Chinese culture and American culture at least differ in one of the five measures we had (SOPVerbal score, SOPVisual score, OSpan working memory capacity, SSpan working memory capacity, and LogAbsNewHI handedness score). Followed-up discriminant analysis showed that among these five measures, OSpan working memory capacity was most able to differentiate the two cultural groups. In other words, Chinese people and American people are distinct in terms of these five measures, and their difference exists mostly in their working memory capacity reflected by the operation span task. Gender might have played a role since the effect was marginally significant, but there was no evidence that the gender differences in the two cultures are distinct.

According to the discriminant function analysis, little of the group difference can be explained by degree of handedness. This finding is not surprising to us. One major reason is that we recruited participants that are representative of the population of college students from the two cultural backgrounds. This rule of recruitment strengthens the generalization of our results, but one side effect is that it also limits the possibility of any other restrictions, including handedness. Thus, most of our participants are strong right-handers which makes it more difficult to detect any handedness-related group or individual difference.

We hypothesized to see a bias toward visual processing style from the Chinese group and one toward verbal from the American group. We did find the American group prefers verbal processing compared to the Chinese group, but the two groups have an equivalent preference to visual processing. One possibility is that the holistic/analytic dichotomy does not necessarily result in a similar one in visualizer/verbalizer; it is also possible that the cultural difference in visualizing/verbalizing is limited to ability and does not transfer to preference. Previous visual research on cultural difference in processing has investigated the ability to either integrate or ignore context information of visual stimuli, and found that Eastern participants are more able to integrate context and focal information while Western participants are more successful ignoring the surroundings when required (Kitayama and Ishii, 2002; Kitayama et al., 2003). East Asians have also been found to have more global advantage (global information processed faster than local information) in Navon task (McKone et al., 2010), suggesting that East Asian’s attention is more directed to the context or global features when encountering visual stimuli (also see Lewis et al., 2008, for supporting evidence from event-related potentials). However, such research has been focused on attention-directed visual processing ability only, and the results do not necessarily mirror individuals’ preferences. Our findings suggest that what affects one’s preference toward daily visual or verbal processing may not be influenced by the holistic/analytic dichotomy like their visualizing ability is. In addition, the fact that the two cultural groups differ in SOPVerbal but SOPVisual also supports our suggestion that verbal and visual SOP should not be treated separately rather than as a unitary bipolar dimension.

The difference in OSpan performance we found is consistent with previous findings concerning math performance in people with unlike cultural backgrounds. One major part of OSpan task is the math problems participants need to solve as a distraction to the working memory task. Participants are required to calculate the answers of addition or subtraction operations while they hold target letters in their working memory. The speed of their operation directly affects how much time they have left before they are presented with the next to-be-remembered letter, therefore influences their rehearsal of previously remembered letters, which relates to their performance in the task. If one is faster in solving mathematical operations, they would be able to have more time to spend on rehearsing the target letters they already remember from the same trial, and end up remembering more letters compared to those who are not as good in math. Previous research has shown that different brain areas are activated when Chinese speakers and English speakers are dealing with math (Tang et al., 2006; Cantlon and Brannon, 2007), and Chinese outperform Americans in numerical cognition even from kindergarten (Geary et al., 1996; Siegler and Mu, 2008). Chinese speakers in the current study could also have outperformed the American group with addition and subtraction operations, and thus performed better in OSpan task, which is exactly what we found with our data. Potential supportive evidence of this possibility is that even both OSpan and SSpan tasks measured working memory capacity, we did not find a similar difference between the two cultural groups with SSpan task.

The fact that cultural difference between Chinese and American group is shown mostly in OSpan but not SSpan performance also supports the claim that in complex span tasks, the working memory capacities they measured are task-specific (Daneman and Carpenter, 1980). This result is consistent with Holmes et al. (2019) study, where they studied whether training benefits transfer from one working memory task to another and found this kind of transfer is constrained by working memory paradigm. Our finding is further supported by ERP research that suggests that a working memory task is composed of a few neural processes, and these processes could be independently affected by the type of task (McEvoy et al., 1998).

Correlation analysis with all participants together showed a significant correlation between OSpan with SSpan, and also that between OSpan and ability in visualizing. From later analysis with the two cultural groups separated, we could see that these correlations mostly came from the American group. It is interesting to see that the variables showed different correlation patterns in Chinese and American cultural groups, as no variables were shown correlated in the Chinese group.

There are some interesting findings from the correlation analysis. Specifically, SSpan performance is significantly correlated with OSpan performance in the American group, but not so in the Chinese group. This could be the consequence of Chinese students’ superior ability in math (for example, as shown by their better performance solving SAT math problems; Byrnes et al., 1997) that also caused the large group difference in OSpan performance. As we discussed before, math ability is vital for solving operation problems, i.e., the secondary task in OSpan, and therefore affects the time and cognitive load left on the primary memory task. Specifically, individuals with better math ability (in our case, the Chinese group) would be able to solve the operations quickly and save time for repeating the to-be-remembered letters in their working memory. In fact, people are found to use rehearsal a lot in working memory tasks, especially OSpan, and rehearsal has been proven to increase OSpan performance (Turley-Ames and Whitfield, 2003; Dunning and Holmes, 2014; however, see Longoni et al., 1993 and Oberauer, 2019 for different opinions). In order to understand whether other aspects of working memory are affected by culture, more manipulation on the tasks is desired in future research, such as the introduction of rehearsal inhibition mechanism.

Another interesting finding from the correlation analysis is that the preference of visual processing is correlated to both working memory span tasks for the American group, but such correlation is not shown with the Chinese group. This finding might be due to the differential involvement of visual processing in their daily language processing. For example, research suggested a significant role of visual encoding in the retention of Chinese words (Shen, 2010). On the contrary, visual learning (visual repetition and imagery mnemonics) is shown to correlate negatively with proficiency in English vocabulary learning (Gu and Johnson, 1996). Encountering with their native language (Chinese and English, respectively) on a daily basis could affect the degree to which performing mental imagery involves the use of working memory, and further influence the correlation between working memory and preference of mental imagery, while most of our SOPVisual problems are about the latter. It is possible that the Chinese group does not rely on a high working memory capacity like their American counterparts to establish a preference toward visual cognitive processing. However, the concrete reason for these correlational relationships needs further investigation.

Besides the culture-related questions we answered with the current study, we also proposed an alternative way of applying some widely-used measures. One is the Edinburgh Handedness Inventory (Oldfield, 1971). The scoring method suggested by Oldfield (1971) emphasizes differentiating left-handers with right-handers and therefore does not precisely reflect the strength of handedness as we wanted. For example, someone who answers “1” to all questions would have exactly the same score as someone who answers “2” to all questions, according to the Oldfield’s scoring method. It is acceptable for studies that care more about the direction of handedness, but the first person should score lower in the strength of being a right-hander as they do not “always” use their right hand in performing the listed actions as the second person does. Our revised scoring method calculates the strength of handedness more accurately so that both degree and direction of handedness can be reflected in one score.

The current study also examined the use of the Style of Processing inventory (SOP). We suggested that in a situation where the research goal is not to tell verbalizers and visualizers apart, but rather how people, respectively, perform in verbal and visual tasks, it is reasonable to separate the questions extract two dimensions from the original SOP score. In this way, we would be able to differentiate people who are good at both types of tasks from those who underperform in both, while having a single score on the visualizer-verbalizer spectrum conceals this difference. This change is conceptually legitimate for the current study. In addition, an increase of internal consistency after separating the items in the inventory provided adequate statistical evidence to support this suggestion.

## Data Availability Statement

The raw data supporting the conclusions of this article will be made available by the authors, without undue reservation.

## Ethics Statement

The studies involving human participants were reviewed and approved by Miami Research Ethics and Integrity Program, Miami University. The patients/participants provided their written informed consent to participate in this study.

## Author Contributions

GF, KC, and RT together conceived the original idea and carried out the experiment. GF and KC collected the data. GF analyzed the data and wrote the manuscript with support from RT. All authors contributed to the article and approved the submitted version.

## Conflict of Interest

The authors declare that the research was conducted in the absence of any commercial or financial relationships that could be construed as a potential conflict of interest.

## References

[B1] Alloway T. P. Alloway R. (2013). *Working Memory: The Connected Intelligence.* New York, NY: Psychology Press.

[B2] Antonietti A. Giorgetti M. (1998). The verbalizer-visualizer questionnaire: a review. *Percept. Motor Skills* 86 227–239. 10.2466/pms.1998.86.1.227 9530739

[B3] Baddeley A. D. Hitch G. (1974). Working memory. *Psychol. Learn. Motiv.* 8 47–89. 10.1016/S0079-7421(08)60452-1

[B4] Barch D. M. Burgess G. C. Harms M. P. Petersen S. E. Schlaggar B. L. Corbetta M. (2013). Function in the human connectome: task-fMRI and individual differences in behavior. *Neuroimage* 80 169–189. 10.1016/j.neuroimage.2013.05.033 23684877 PMC4011498

[B5] Barrouillet P. Portrat S. Camos V. (2011). On the law relating processing to storage in working memory. *Psychol. Rev.* 118:175. 10.1037/a0022324 21480738

[B6] Blajenkova O. Kozhevnikov M. Motes M. A. (2006). Object-spatial imagery: a new self-report imagery questionnaire. *Appl. Cogn. Psychol.* 20 239–263. 10.1002/acp.1182

[B7] Blazhenkova O. Kozhevnikov M. (2009). The new object-spatial-verbal cognitive style model: theory and measurement. *Appl. Cogn. Psychol.* 23 638–663. 10.1002/acp.1473

[B8] Bloom J. S. Hynd G. W. (2005). The role of the corpus callosum in interhemispheric transfer of information: excitation or inhibition? *Neuropsychol. Rev.* 15 59–71. 10.1007/s11065-005-6252-y 16211466

[B9] Brislin R. W. (1970). Back-translation for cross-cultural research. *J. Cross Cult. Psychol.* 1 185–216. 10.1177/135910457000100301

[B10] Bryden M. P. (1987). “Handedness and cerebral organization: data from clinical and normal populations,” in *Duality and Unity of the Brain*, ed. Ottoson D. (Houndmills: Macmillan Publishing Co Inc), 55–70. 10.1007/978-1-349-08940-6_5

[B11] Byrnes J. P. Hong L. Xing S. (1997). Gender differences on the math subtest of the Scholastic Aptitude Test may be culture-specific. *Educ. Stud. Math.* 34 49–66.

[B12] Byun S. Y. Park H. (2012). The academic success of East Asian American youth: the role of shadow education. *Sociol. Educ.* 85 40–60. 10.1177/0038040711417009 24163483 PMC3806291

[B13] Campbell J. R. Mandel F. (1990). Connecting math achievement to parental influences. *Contemp. Educ. Psychol.* 15 64–74. 10.1016/0361-476x(90)90006-m

[B14] Cantlon J. F. Brannon E. M. (2007). Adding up the effects of cultural experience on the brain. *Trends Cogn. Sci.* 11 1–4. 10.1016/j.tics.2006.10.008 17129750

[B15] Caplan P. J. Crawford M. Hyde J. S. Richardson J. T. (1997). *Gender Differences in Human Cognition. Counterpoints: Cognition, Memory, and Language Series.* Cary, NC: Oxford University Press.

[B16] Chen C. Stevenson H. W. (1995). Motivation and mathematics achievement: a comparative study of Asian-American, Caucasian-American, and East Asian high school students. *Child Dev.* 66 1215–1234. 10.2307/1131808 7671657

[B17] Childers T. L. Houston M. J. Heckler S. E. (1985). Measurement of individual differences in visual versus verbal information processing. *J. Consum. Res.* 12 125–134. 10.1086/208501

[B18] Chow M. Conway A. R. A. (2015). The scope and control of attention: sources of variance in working memory capacity. *Mem. Cogn.* 43 325–339. 10.3758/s13421-014-0496-9 25604642

[B19] Christman S. D. Propper R. E. (2010). “Episodic memory and interhemispheric interaction: handedness and eye movements,” in *Current Issues in Applied Memory Research*, eds Davies G. M. Wright D. B. (New York, NY: Psychology Press), 185–205.

[B20] Christman S. D. Sontam V. Jasper J. D. (2009). Individual differences in ambiguous figure perception: degree of handedness and interhemispheric interaction. *Perception* 38 1183–1198. 10.1068/p6131 19817151

[B21] Clarke J. M. Zaidel E. (1994). Anatomical-behavioral relationships: corpus callosum morphometry and hemispheric specialization. *Behav. Brain Res.* 64 185–202. 10.1016/0166-4328(94)90131-7 7840886

[B22] Collins R. L. (1985). “On the inheritance of direction and degree of asymmetry,” in *Cerebral Lateralization in Nonhuman Species*, ed. Glick S. (New York, NY: Academic Press), 41–71. 10.1016/b978-0-12-286480-3.50009-4

[B23] Conway A. R. Kane M. J. Bunting M. F. Hambrick D. Z. Wilhelm O. Engle R. W. (2005). Working memory span tasks: a methodological review and user’s guide. *Psychon. Bull. Rev.* 12 769–786. 10.3758/BF03196772 16523997

[B24] Cook D. A. (2008). Scores from Riding’s cognitive styles analysis have poor test–retest reliability. *Teaching Learn. Med.* 20 225–229. 10.1080/10401330802199492 18615296

[B25] Cowan N. (2010). The magical mystery four how is working memory capacity limited, and why? *Curr. Dir. Psychol. Sci.* 19 51–57. 10.1177/0963721409359277 20445769 PMC2864034

[B26] Crinion J. T. Green D. W. Chung R. Ali N. Grogan A. Price G. R. (2009). Neuroanatomical markers of speaking Chinese. *Hum. Brain Mapp.* 30 4108–4115. 10.1002/hbm.20832 19530216 PMC3261379

[B27] Daneman M. Carpenter P. A. (1980). Individual differences in working memory and reading. *J. Verbal Learn. Verbal Behav.* 19 450–466. 10.1016/S0022-5371(80)90312-6

[B28] de Fockert J. W. Rees G. Frith C. D. Lavie N. (2001). The role of working memory in visual selective attention. *Science* 291 1803–1806. 10.1126/science.1056496 11230699

[B29] Deniz F. Nunez-Elizalde A. O. Huth A. G. Gallant J. L. (2019). The representation of semantic information across human cerebral cortex during listening versus reading is invariant to stimulus modality. *J. Neurosci.* 39 7722–7736. 10.1523/JNEUROSCI.0675-19.2019 31427396 PMC6764208

[B30] Downing P. E. (2000). Interactions between visual working memory and selective attention. *Psychol. Sci.* 11 467–473. 10.1111/1467-9280.00290 11202491

[B31] Dunning D. L. Holmes J. (2014). Does working memory training promote the use of strategies on untrained working memory tasks? *Memory & Cognition*, 42 854–862. 10.3758/s13421-014-0410-5 24748348 PMC4110412

[B32] Engle R. W. Tuholski S. W. Laughlin J. E. Conway A. R. (1999). Working memory, short-term memory, and general fluid intelligence: a latent-variable approach. *J. Exp. Psychol. Gen.* 128 309. 10.1037/0096-3445.128.3.309 10513398

[B33] Erickson K. I. Voss M. W. Prakash R. S. Basak C. Szabo A. Chaddock L. (2011). Exercise training increases size of hippocampus and improves memory. *Proc. Natl. Acad. Sci. U.S.A.* 108 3017–3022. 10.1073/pnas.1015950108 21282661 PMC3041121

[B34] Frolov A. A. Mokienko O. Lyukmanov R. Biryukova E. Kotov S. Turbina L. (2017). Post-stroke rehabilitation training with a motor-imagery-based brain-computer interface (BCI)-controlled hand exoskeleton: a randomized controlled multicenter trial. *Front. Neurosci.* 11:400. 10.3389/fnins.2017.00400 28775677 PMC5517482

[B35] Geary D. C. Bow-Thomas C. C. Liu F. Siegler R. S. (1996). Development of arithmetical competencies in Chinese and American children: influence of age, language, and schooling. *Child Dev.* 67 2022–2044. 10.1111/j.1467-8624.1996.tb01841.x 9022227

[B36] Gu Y. Johnson R. K. (1996). Vocabulary learning strategies and language learning outcomes. *Lang. Learn.* 46 643–679. 10.1111/j.1467-1770.1996.tb01355.x

[B37] Hardie S. M. Wright L. (2014). Differences between left- and right-handers in approach/avoidance motivation: influence of consistency of handedness measures. *Front. Psychol.* 5:134. 10.3389/fpsyg.2014.00134 24600426 PMC3929948

[B38] Heckler S. E. Childers T. L. Houston M. J. (1993). On the construct validity of the SOP scale. *J. Ment. Imag.* 17 119–132.

[B39] Hellige J. B. (2001). *Hemispheric Asymmetry: What’s Right and What’s Left.* Cambridge, MA: Harvard University Press.

[B40] Holmes J. Woolgar F. Hampshire A. Gathercole S. E. (2019). Are working memory training effects paradigm-specific? *Front. Psychol.* 10:1103. 10.3389/fpsyg.2019.01103 31178781 PMC6542987

[B41] Hu Y. H. Qiou Y. G. Zhong G. Q. (1990). Crossed aphasia in Chinese: a clinical survey. *Brain Lang.* 39 347–356. 10.1016/0093-934X(90)90144-6 1704810

[B42] Imbo I. LeFevre J. A. (2010). The role of phonological and visual working memory in complex arithmetic for Chinese-and Canadian-educated adults. *Mem. Cogn.* 38 176–185. 10.3758/MC.38.2.176 20173190

[B43] Isaacs K. L. Barr W. B. Nelson P. K. Devinsky O. (2006). Degree of handedness and cerebral dominance. *Neurology* 66 1855–1858. 10.1212/01.wnl.0000219623.28769.74 16801650

[B44] Ismatullina V. Voronin I. Shelemetieva A. Malykh S. (2014). Cross-cultural study of working memory in adolescents. *Procedia Soc. Behav. Sci.* 146 353–357. 10.1016/j.sbspro.2014.08.111

[B45] Ivry R. B. Robertson L. C. (1998). *The Two Sides of Perception.* Cambridge, MA: The MIT Press.

[B46] Josse G. Tzourio-Mazoyer N. (2004). Hemispheric specialization for language. *Brain Res. Rev.* 44 1–12. 10.1016/j.brainresrev.2003.10.001 14739000

[B47] Kane M. J. Hambrick D. Z. Tuholski S. W. Wilhelm O. Payne T. W. Engle R. W. (2004). The generality of working memory capacity: a latent-variable approach to verbal and visuospatial memory span and reasoning. *J. Exp. Psychol. Gen.* 133:189. 10.1037/0096-3445.133.2.189 15149250

[B48] Kitayama S. Ishii K. (2002). Word and voice: spontaneous attention to emotional utterances in two languages. *Cogn. Emot.* 16 29–59. 10.1080/0269993943000121

[B49] Kitayama S. Duffy S. Kawamura T. Larsen J. T. (2003). Perceiving an object and its context in different cultures: a cultural look at new look. *Psychol. Sci.* 14 201–206. 10.1111/1467-9280.02432 12741741

[B50] Kitterle F. L. Christman S. Conesa J. S. (1995). “Spatial-frequency selectivity in hemispheric transfer,” in *Hemispheric Communication: Mechanisms and Models*, ed. Kitterle F. L. (Hillsdale, NJ: Lawrence Erlbaum Associates), 319–345. 10.4324/9781315789156-11

[B51] Kiyokawa S. Dienes Z. Tanaka D. Yamada A. Crowe L. (2012). Cross cultural differences in unconscious knowledge. *Cognition* 124 16–24. 10.1016/j.cognition.2012.03.009 22560768

[B52] Kochunov P. Fox P. Lancaster J. Tan L. H. Amunts K. Zilles K. (2003). Localized morphological brain differences between English-speaking Caucasians and Chinese-speaking Asians: new evidence of anatomical plasticity. *Neuroreport* 14 961–964. 10.1097/01.wnr.0000075417.59944.00 12802183

[B53] Kozhevnikov M. (2007). Cognitive styles in the context of modern psychology: toward an integrated framework of cognitive style. *Psychol. Bull.* 133 464–481. 10.1037/0033-2909.133.3.464 17469987

[B54] Lee K. M. Kang S. Y. (2002). Arithmetic operation and working memory: differential suppression in dual tasks. *Cognition* 83 B63–B68. 10.1016/S0010-0277(02)00010-0 11934408

[B55] Lewis R. S. Goto S. G. Kong L. L. (2008). Culture and context: east Asian American and European American differences in P3 event-related potentials and self-construal. *Pers. Soc. Psychol. Bull.* 34 623–634. 10.1177/0146167207313731 18413894

[B56] Longoni A. M. Richardson J. T. Aiello A. (1993). Articulatory rehearsal and phonological storage in working memory. *Mem. Cogn.* 21 11–22. 10.3758/BF03211160 8433641

[B57] Masuda T. Nisbett R. E. (2001). Attending holistically versus analytically: comparing the context sensitivity of Japanese and Americans. *J. Pers. Soc. Psychol.* 81:922. 10.1037/0022-3514.81.5.922 11708567

[B58] Mayer R. E. Massa L. J. (2003). Three facets of visual and verbal learners: cognitive ability, cognitive style, and learning preference. *J. Educ. Psychol.* 95 833–846. 10.1037/0022-0663.95.4.833

[B59] McEvoy L. K. Smith M. E. Gevins A. (1998). Dynamic cortical networks of verbal and spatial working memory: effects of memory load and task practice. *Cereb. Cortex (New York NY 1991)* 8 563–574. 10.1093/cercor/8.7.563 9823478

[B60] McKone E. Davies A. A. Fernando D. Aalders R. Leung H. Wickramariyaratne T. (2010). Asia has the global advantage: race and visual attention. *Vis. Res.* 50 1540–1549. 10.1016/j.visres.2010.05.010 20488198

[B61] Na J. Chan M. Y. (2015). “Culture, cognition, and intercultural relations,” in *Neuroscience in Intercultural Contexts*, eds Warnick J. E. Landis D. (New York, NY: Springer), 49–71. 10.1007/978-1-4939-2260-4_3

[B62] Newman S. Malaia E. Seo R. (2014). Does degree of handedness in a group of right-handed individuals affect language comprehension? *Brain Cogn.* 86 98–103. 10.1016/j.bandc.2014.02.002 24607732 PMC4006107

[B63] Nisbett R. E. Masuda T. (2003). Culture and point of view. *Proc. Natl. Acad. Sci. U.S.A.* 100 11163–11170. 10.1073/pnas.1934527100 12960375 PMC196945

[B64] Nisbett R. E. Peng K. Choi I. Norenzayan A. (2001). Culture and systems of thought: holistic versus analytic cognition. *Psychol. Rev.* 108:291. 10.1037/0033-295X.108.2.291 11381831

[B65] Oberauer K. (2019). Is rehearsal an effective maintenance strategy for working memory? *Trends Cogn. Sci.* 23 798–809. 10.1016/j.tics.2019.06.002 31301953

[B66] Oldfield R. C. (1971). The assessment and analysis of handedness and Edinburgh inventory. *Neuropsychologia* 9 97–113. 10.1016/0028-3932(71)90067-4 5146491

[B67] Ong Y. W. Milech D. (2001). The style of processing scale: normative and reliability data. *Percept. Motor Skills* 93 595–598. 10.2466/pms.2001.93.3.595 11806573

[B68] Paivio A. (1971). *Imagery and Verbal Processes.* New York, NY: Holt, Rinehart & Winston.

[B69] Prichard E. Propper R. E. Christman S. D. (2013). Degree of handedness, but not direction, is a systematic predictor of cognitive performance. *Front. Psychol.* 4:9. 10.3389/fpsyg.2013.00009 23386836 PMC3560368

[B70] Pujol J. Deus J. Losilla J. M. Capdevila A. (1999). Cerebral lateralization of language in normal left-handed people studied by functional MRI. *Neurology* 52 1038–1038. 10.1212/WNL.52.5.1038 10102425

[B71] Redick T. S. Broadway J. M. Meier M. E. Kuriakose P. S. Unsworth N. Kane M. J. (2012). Measuring working memory capacity with automated complex span tasks. *Eur. J. Psychol. Assess.* 28:164. 10.1027/1015-5759/a000123

[B72] Richardson A. (1977). Verbalizer-Visualizer: A cognitive style dimension. *Journal of Mental Imagery*, 1 109–126.

[B73] Riding R. J. (1997). On the nature of cognitive style. *Educ. Psychol.* 17 29–49. 10.1080/0144341970170102

[B74] Riding R. Cheema I. (1991). Cognitive styles—an overview and integration. *Educational Psychology*, 11 193–215. 10.1080/0144341910110301

[B75] Riding R. Rayner S. (Eds.) (1998). *Cognitive Styles and Learning Strategies: Understanding style Differences in Learning and Behaviour.* London: David Fulton Publishers.

[B76] Schlaug G. Jäncke L. Huang Y. Staiger J. F. Steinmetz H. (1995). Increased corpus callosum size in musicians. *Neuropsychologia* 33 1047–1055. 10.1016/0028-3932(95)00045-5 8524453

[B77] Schmitt B. H. Pan Y. Tavassoli N. T. (1994). Language and consumer memory: the impact of linguistic differences between Chinese and English. *J. Consum. Res.* 21 419–431. 10.1086/209408

[B78] Shen H. H. (2010). Imagery and verbal coding approaches in Chinese vocabulary instruction. *Lang. Teach. Res.* 14 485–499. 10.1177/1362168810375370

[B79] Siegler R. S. Mu Y. (2008). Chinese children excel on novel mathematics problems even before elementary school. *Psychol. Sci.* 19 759–763. 10.1111/j.1467-9280.2008.02153.x 18816281

[B80] Siok W. T. Perfetti C. A. Jin Z. Tan L. H. (2004). Biological abnormality of impaired reading is constrained by culture. *Nature* 431 71–76. 10.1038/nature02865 15343334

[B81] Springer S. P. Deutsch G. (1985). *Left Brain, Right Brain: Perspectives on Cognitive Neuroscience.* New York: Freeman.

[B82] Stevenson H. W. Lee S. Y. Chen C. Stigler J. W. Hsu C. C. Kitamura S. (1990). Contexts of achievement: a study of American, Chinese, and Japanese children. *Monogr. Soc. Res. Child Dev.* 55 1–123. 2342493

[B83] Stoodley C. J. Valera E. M. Schmahmann J. D. (2012). Functional topography of the cerebellum for motor and cognitive tasks: an fMRI study. *Neuroimage* 59 1560–1570. 10.1016/j.neuroimage.2011.08.065 21907811 PMC3230671

[B84] Tan L. H. Liu H. L. Perfetti C. A. Spinks J. A. Fox P. T. Gao J. H. (2001). The neural system underlying Chinese logograph reading. *Neuroimage* 13 836–846. 10.1006/nimg.2001.0749 11304080

[B85] Tan L. H. Spinks J. A. Gao J. H. Liu H. L. Perfetti C. A. Xiong J. (2000). Brain activation in the processing of Chinese characters and words: a functional MRI study. *Hum. Brain Mapp.* 10 16–27. 10.1002/(sici)1097-0193(200005)10:1<16::aid-hbm30>3.0.co;2-m 10843515 PMC6871809

[B86] Tang Y. Zhang W. Chen K. Feng S. Ji Y. Shen J. (2006). Arithmetic processing in the brain shaped by cultures. *Proc. Natl. Acad. Sci. U.S.A.* 103 10775–10780. 10.1073/pnas.0604416103 16815966 PMC1502307

[B87] Turley-Ames K. J. Whitfield M. M. (2003). Strategy training and working memory task performance. *J. Mem. Lang.* 49 446–468. 10.1016/S0749-596X(03)00095-0

[B88] Turner M. L. Engle R. W. (1989). Is working memory capacity task dependent? *J. Mem. Lang.* 28 127–154. 10.1016/0749-596X(89)90040-5

[B89] Unsworth N. Engle R. W. (2007). On the division of short-term and working memory: an examination of simple and complex span and their relation to higher order abilities. *Psychol. Bull.* 133:1038. 10.1037/0033-2909.133.6.1038 17967093

[B90] Unsworth N. Heitz R. Schrock J. Engle R. (2005). An automated version of the operation span task. *Behav. Res. Methods* 37 498–505. 10.3758/BF03192720 16405146

[B91] Voss M. W. Prakash R. S. Erickson K. I. Basak C. Chaddock L. Kim J. S. (2010). Plasticity of brain networks in a randomized intervention trial of exercise training in older adults. *Front. Aging Neurosci.* 2:32. 10.3389/fnagi.2010.00032 20890449 PMC2947936

[B92] Whang P. A. Hancock G. R. (1994). Motivation and mathematics achievement: comparisons between Asian-American and non-Asian students. *Contemp. Educ. Psychol.* 19 302–322. 10.1006/ceps.1994.1023

[B93] Witelson S. F. (1985). The brain connection: the corpus callosum is larger in left-handers. *Science* 229 665–668. 10.1126/science.4023705 4023705

[B94] Witkin H. A. (1971). *A Manual for the Embedded Figures Tests.* Palo Alto, CA: Consulting Psychologists Press.

[B95] Witkin H. A. Goodenough D. R. (1981). *Cognitive Styles, Essences and Origins: Field Dependence and Field Independence.* New York, NY: International Universities. 7012884

[B96] Yamaguchi S. Yamagata S. Kobayashi S. (2000). Cerebral asymmetry of the top-down allocation of attention to global and local features. *J. Neurosci.* 20:RC72. 10.1523/JNEUROSCI.20-09-j0002.2000 10777814 PMC6773123

